# Minimally Invasive Video-Assisted Thyroidectomy and Parathyroidectomy with Intraoperative Recurrent Laryngeal Nerve Monitoring

**DOI:** 10.1155/2010/834913

**Published:** 2010-06-06

**Authors:** Gianlorenzo Dionigi, Luigi Boni, Stefano Rausei

**Affiliations:** Department of Surgical Sciences, University of Insubria (Como-Varese), Ospedale di Circolo-Polo Universitario, Viale Borri 57, 21100 Varese, Italy

Dr. Kandil [1] reported their experience in intraoperative neuromonitoring (IONM) in endoscopic thyroid surgery. I have carefully read this paper. These colleagues have made a similar contribution in respect of our previous paper (published online on Surgical Endoscopy on September 21, 2008) [2]. 

I would like to point out that a comparative series on endoscopic thyroidectomy with IONM versus no use of IONM have been already and equally proposed in the English Literature with “*emphasis given to the identification of recurrent laryngeal nerve (RLN)” *and external branch of superior laryngeal nerve (EBSLN) [2]. This study was based on a prospectively randomized series comprising 72 standard VAT gasless approaches. In the control group (*N* = 36), the laryngeal nerves were identified by 30-degree 5 mm endoscope magnification solely. Likewise “*there was no instance of equipment malfunction or interference”* [2]. No permanent complications occurred in either group. More precise technical details on MIVAT and IONM are exposed in our paper [2]. The incidences of temporary RLN injury were 2.7% and 8.3% in the IONM and control group, respectively [2]. The EBSLN was identified better in the IONM group: 83.6% versus 42% (*P*  <  .05) [2]. In our paper we conclude equally that “*neuromonitoring enables surgeons to feel more comfortable with MIVAT”* [2]. Other similar conclusions are exposed in the discussion section [1, 2].

There is a base technical issue that we would like to comment and underline. Dr. Kandil reported a “*standardized IONM technique”*. Actually, Dr. Kandil stimulated the RLN and not the vagal nerve. The standardization of IONM technique covers a fundamental technical aspect in thyroid surgery also in endoscopic thyroidectomy [2–7]. Stimulation via the vagal nerve is essential to recognize any RLN lesions and to predict nerve postoperative function: in neurogenic lesions of the RLN distal stimulation near the larynx produces a false negative, “normal” IONM signal [2–7]. Only vagal stimulation and, in addition, electromyographic (EMG) registration of signals, which easily uncovers all kinds of artifacts, can help avoid spurious EMG findings and clarify the real impact of IONM on thyroidectomy [2–4]. This is in concordance with Chiang, Timmermann, and Dralle remarks [2–7]. 

Technically, the vagus nerve is stimulated directly by dissecting the carotid sheath just from a 1 cm pouch or in some cases only by simply applying the stimulator on the carotid sheath without dissection (usually in patients with low fat in the neck) [2–7]. Vagal stimulation does not result in increasing in morbidity, surgical incision length, or operative time [2]. Finally, vagal nerve stimulation permits to confirm RLN function when visual identification of the RLN is very difficult or hazardous [6]. 

*Gianlorenzo Dionigi* *Gianlorenzo Dionigi* *Luigi Boni* *Luigi Boni* *Stefano Rausei* *Stefano Rausei*
